# Dynamic time warp of emotions in patients with cutaneous T-cell lymphoma treated with corticosteroids

**DOI:** 10.1016/j.jdin.2024.07.015

**Published:** 2024-08-23

**Authors:** Anne-Sophie C.A.M. Koning, Rosanne Ottevanger, Maarten H. Vermeer, Onno C. Meijer, Erik J. Giltay

**Affiliations:** a Department of Medicine, Division of Endocrinology, Leiden University Medical Center, Leiden, the Netherlands; b Department of Dermatology, Leiden University Medical Center, Leiden, the Netherlands; c Department of Psychiatry, Leiden University Medical Center, Leiden, the Netherlands; d Department of Public Health and Primary Care, Health Campus the Hague, Leiden University Medical Center, the Hague, the Netherlands

**Keywords:** dynamic time warping, emotions, glucocorticoids, network analysis, time-series

## Abstract

**Background:**

A substantial number of patients treated systemically with synthetic glucocorticoids undergo emotional disturbances during treatment. Patients with cutaneous T-cell lymphoma frequently experience skin inflammation and itching and often require glucocorticoid treatment.

**Objective:**

This case-series study aimed to examine how emotional and skin-related symptoms interact throughout glucocorticoid treatment.

**Methods:**

Five cutaneous T-cell lymphoma patients undergoing systemic glucocorticoid treatment completed daily ecological momentary assessments for on average 30 assessments. Fluctuations in their emotions and symptoms were analyzed using undirected and directed dynamic time warp analyses, and were visualized in symptom networks.

**Results:**

Toward the end of the glucocorticoid treatment, a decline was found in positive psychological symptoms. Idiographic dynamic time warp analyses revealed highly variable symptom networks. Directed time-lag group-level analyses revealed irritability, enthusiastic, and excited as variables with highest outstrength, in which mainly decreasing levels of positive emotions were associated with a higher likelihood of experiencing increases in itchy skin and skin problems the next day.

**Conclusion:**

The end of glucocorticoid treatment, potentially via the induction of hypocortisolism, seems to coincide with decreased energy, motivation, and enthusiasm. Itch and skin problems could be a consequence of low-positive emotions the day before.

**Capsule Summary**

• Dynamic time warping enhances our understanding on how emotions and symptoms influence each other over time.
• Group-level analyses showed that increased irritability and low levels of positive emotions were followed by increased skin problems and itchy skin the next day. Individual analyses demonstrated large variability, suggesting potential for personalized treatment.

## Introduction

It is well-known that the use of synthetic glucocorticoids can result in neuropsychiatric adverse effects, such as depression, anxiety, mania, delirium, psychosis, sleep disturbances, and even suicidality.^1^^,^^2^ Although not all patients experience neuropsychiatric adverse effects, many do feel different and undergo some emotional disturbances, including feelings of restlessness, irritability, stress, mild euphoria, and sleep problems during treatment.^3^, ^4^, ^5^ These symptoms are often relatively mild and patients may not actively seek treatment, but they can still adversely affect the patient’s quality of life. Glucocorticoids are used for their potent anti-inflammatory effects in various medical conditions, including immune diseases and different types of cancer.^6^, ^7^, ^8^ A notable example is cutaneous T-cell lymphoma (CTCL), a rare heterogeneous group of T-cell lymphomas that primarily manifest in the skin with diverse manifestations. These result in symptoms ranging from mild to very severe that degrade the patient’s quality of life. CTCL has scarce curative options and because of the chronic course, therapy that suppresses symptoms is often required.^9^, ^10^, ^11^ Despite the development of novel therapies, such as CCR4 antibody therapies,^12^ synthetic glucocorticoids are still frequently used systemically, especially when symptoms flareup severely. Their effects on symptoms and emotions in this specific group of patients have not been thoroughly investigated thus far.

Indeed, to our knowledge, no studies have employed a symptom network approach to investigate effects of (or during) glucocorticoid treatment. In the current study, we used a network approach in which the temporal dynamics of emotions and skin problems were investigated. Temporal dynamics of diverse variables such as disease symptoms and emotions can be explored through the analyses of time-series data, such as with dynamic time warp (DTW). DTW is a relatively new analytical method in which the temporal dynamics of time series can be analyzed first within participants (idiographic approach), and after aggregation also at the group-level (nomothetic approach). The fact that both approaches can be performed is important as group-derived estimates are often not accurate proxies of individual processes.^13^ DTW has already been used in depression and bipolar disorder research, and it demonstrated that symptom dynamics or trajectories over time can be captured and visualized in network plots.^14^, ^15^, ^16^, ^17^, ^18^, ^19^

Utilizing DTW, we aimed to gain deeper insights into the dynamics of emotions and symptoms during glucocorticoid treatment both at an individual level and group-level. For these analyses, we used ecological momentary assessment data from 5 patients with CTCL during a glucocorticoid treatment. We hypothesize that the idiographic approach will show variability among individual networks. Second, with the nomothetic approach we expect to identify some key emotions within the network that tend to trigger other emotions or symptoms. Such findings may help to design better supportive interventions, counseling, or pharmacologic interventions targeting mood regulation to improve overall treatment experience of individual patients.

## Methods

### Study design

This study was a prospective observational pilot study, conducted at the Department of Dermatology at the Leiden University Medical Center in the Netherlands between September 2022 and April 2023. The study is performed in accordance with the principles of the revised Declaration of Helsinki. Written informed consent was obtained from all participants. The study was registered at ClinicalTrials.gov (NCT05391295).

### Study population

We included 5 patients with mycosis fungoides, the most frequent type of CTCL, who were going to receive oral prednisone treatment. The prednisone treatment was intended as a boost to quickly suppress the skin complaints and starting additional treatment afterward. The glucocorticoid treatment prescribed was tapered every week with a starting dose of 15 to 20 mg daily in the first week for a duration of 30-days with a varying reduction schedule. For study participation, no specific inclusion criteria were imposed, except Dutch speaking because the study was conducted in Dutch, and an age of ≥16 years for informed consent purposes.

### Procedure

Patients underwent evaluations with a dermatologist during outpatient clinic appointments. Dermatologists assessed whether to initiate glucocorticoid treatment. Patients who were prescribed glucocorticoids and started their treatment were eligible for study participation. The treating physician provided oral and written study information. After providing written consent, the participants received assistance with the installation of the m-Path app (www.m-Path.io) on their personal smartphones. The m-Path app serves as a platform for ecological momentary assessments, which can repeatedly deliver short questionnaires.^20^ In our study, participants were asked to complete emotion and symptom-based questions in the application once a day during their glucocorticoid treatment regime. At the designated time of 8:00 PM, M-path generated an alarm beep at 8:00 PM to prompt participants to complete the questionnaire on their smartphones. They were instructed to do so immediately after the beep, but a reminder beep was generated at 9:00 PM to ensure completion of the questionnaire if necessary.

### Measures

The questionnaire consisted of 21 emotion-oriented questions and 2 disease-specific questions. The emotion questions included: feeling good, feeling happy, having sleep problems, feeling relaxed, feeling upset, feeling excited, feeling irritability, feeling motivated, feeling sad, feeling energetic, feeling enthusiastic, feeling nervous, feeling bored, feeling calm, feeling anxious, feeling guilty, having a good concentration, feeling hopeful, feeling tired, experiencing physical discomfort, and worrying. The 2 disease-specific questions included: to what extent are you experiencing symptoms of your skin disease at this time? and to what extent do you experience itching at this time? All questions were answered by means of a slider, and with some questions utilizing a slider response with animated smiley faces. The continuous slider scale ranged from 0 to 100.

### Statistical analysis

The demographic and clinical characteristics were described by means and ranges. The average level of emotions and symptoms during the glucocorticoid treatment were estimated by fitted generalized additive models. Generalized additive models are a flexible extension of traditional linear models that allow for nonlinear relationships between variables. These models can capture complex (both linear and nonlinear effects) patterns and trends in the data by incorporating smooth functions of predictor variables.

DTW was used to calculate similarities of symptom dynamics within participants, both in undirected and directed analyses. DTW is a shape-based time-series clustering technique, which calculates the distance between 2 time series. Meaning that when symptoms behave similar over time (ie, similar dynamics), the distance between symptoms will be small. On the other hand, when symptoms behave different over time the distance will be large.

Within each subject (ie, idiographic approach), we used DTW to calculate each distance between each pair of symptoms based on the optimum warping path between 2 series. The distance calculations utilize a cost matrix, where the DTW algorithm finds a path that minimizes the alignment between the 2 time series. By starting in the left lower corner and ends in the upper right corner, takes steps in the direction with the least increase in cost distance, yielding the total distance score. In Koning et al^19^ detailed information on DTW analysis can be found.

Some constraints were set for DTW. For the undirected analyses, we used the step pattern “symmetric2” and the global constraint of a “Sakoe-Chiba” window size of 1 around main diagonal in the cost matrix. With this window only changes in symptoms that occur between *t* – 1 or *t* + 1 time point away were used for warping (Giorgino et al^21^). Dissimilar scores at the start and end of each panel data could have a disproportional effect on the total distance, because these cannot be dynamically aligned. To reduce this potential distorting effect, we used interpolation of 5 values between each time point before calculating the distance, which consequently reduced the potentially disrupting effect of matching the starting and end point between the 2 time series. A hierarchical cluster analysis was applied according to “Ward.D2” clustering methods, and visualized in a dendrogram that shows dimensions of the emotion items. It is expected that similar emotions will fall in the same dimension.

The idiographic approach yielded in 5 individual distance matrices, one for each participant. We conducted *t* tests to compare the average distance between each pair of symptoms against all other symptom distances. If the distance between a symptom pair was significantly smaller (as determined by a *P* < .05), an edge was displayed between these symptoms in the group-level network plot, following a nomothetic approach. This represented the stable part in the similarities of the dynamics of the symptoms between participants.

For the directed analyses, we used the same DTW algorithm as before, with one crucial difference: the window type was set to be asymmetric, based on a modified version of the “Sakoe-Chiba” band. This adjustment allowed for dynamic alignment to occur exclusively in a forward direction in time. The final distance is determined by the positive relative difference between those 2 distances, namely the distance from symptom A to B versus the distance from B to A. The higher this outcome is, the stronger the temporal effect of one item to another. The 5 distance matrices were subsequently analyzed on the group-level for the directed analysis (ie, nomothetic approach) that yielded the stable part in the similarities of the dynamics of the symptoms between participants. In the directed network plot, arrows were used to represent only those average distances that were significantly different from 0 (as determined by a *P* < .05).

With the directed and undirected matrices network plots were plotted for each individual, and at the group-level. In all network plots the size of the node is proportional to the connectivity of that node. As for the edges, the darkness and thickness are proportional to the strength of the effect. Items with similar dynamics over time have the best alignment with the smallest distance, resulting in the strongest edges in the network plots. In directed network plots, the edges are represented by arrows that show the temporal direction. In addition, the instrength and outstrength centrality was derived: the number/strength of incoming edges of a specific node, and the number/strength of outgoing edges from a specific node, respectively. In our DTW analysis, high outstrength for a specific symptom would imply that changes in this symptom preceded changes in other symptoms. High instrength would imply that changes in this symptom tended to follow upon changes in other symptoms.

The r-packages “mgcv” (version 1.8-38), “tidyverse” (version 1.3.2), “qgraph” (version 1.9.2), “lme4” (version 1.1-30), “dtw” (version 1.23-1), and “parallelDist” (version 0.2.6) for RStudio statistical software were used (R version 4.2.0; R Foundation for Statistical Computing, https://www.R-project.org/). For further reading, other studies have previously used the DTW approach.^14^, ^15^, ^16^, ^17^, ^18^, ^19^

## Results

### Group characteristics

Five patients were included. The mean age of the participants was 53 (range, 21-62 years) years, of whom 2 were women. The mean number of days that the participants completed the assessments was 30 (range, 13-51). Three of the 5 participants followed the prescribed glucocorticoid treatment with a reduction schedule, and 1 continued to use 1 × 15 mg daily during all 4 weeks. One patient discontinued treatment after a few days because of improvement of symptoms and mild side effects. For 2 patients the prednisone treatment course was insufficient and for one of them another treatment was initiated. For 1 patient a prednisone tapering course was initiated after 4 weeks of continued 1 × 15 mg usage, and for 2 patients the prednisone was sufficient for their skin symptoms. Four out of 5 participants filled out (most of) the daily questionnaires. For all participants, it was the first time they received the oral prednisone treatment and they all used the dermal cream clobetasol (once a day, 5 days per week). In Table I the participants’ characteristics are shown.

**Table I:** Participant characteristics

Participants	Daily treatment received	Comorbidity	Other medication	The course
Participant 1	Week 1: 1 × 20 mg Week 2: 1 × 15 mg Week 3: 1 × 10 mg Week 4: 1 × 5 mg	Hypertension	Interferon-α Clobetasol cream	Insufficient.
Participant 2	Week 1, 2: 1 × 20 mg Week 3: 1 × 10 mg	Hypertension Type 2 diabetes	Acitretin (started with the prednisone) Hydroxyzine Clobetasol cream Metformin Metoprolol Simvastatin	First 2 wk Sufficient, during tapering the itch increased: UV-B light therapy (in combination with acitretin) was started after the prednisone treatment.
Participant 3	60 d: 1 × 15 mg	Depression	Trimethoprim-sulfamethoxazole Calcium carbonate Alendronic acid Paroxetine Clobetasol cream	Sufficient, starting a 4 wk tapering course after 60-d 1 × 15 mg course.
Participant 4	Week 1: 1 × 20 mg Week 2: 1 × 20 mg Week 3: 1 × 10 mg Week 4: 1 × 10 mg	Type 2 diabetes Atrial fibrillation Hernia	Methotrexate Folate Clobetasol cream Gliclazide Metformin Pradaxa Amlodipine Losartan Metoprolol Atorvastatin	Sufficient. Skin problems improved with prednisone treatment.
Participant 5	Day 1, 2, 3: 1 × 15 mg Day 4 starting 1 × 10 mg for a few days (exact days not known)	Eating disorder	Clobetasol cream	Participant stopped the treatment, because skin complaints improved and due to insomnia.

### Average level of emotions and symptoms over time

**Fig 1:**
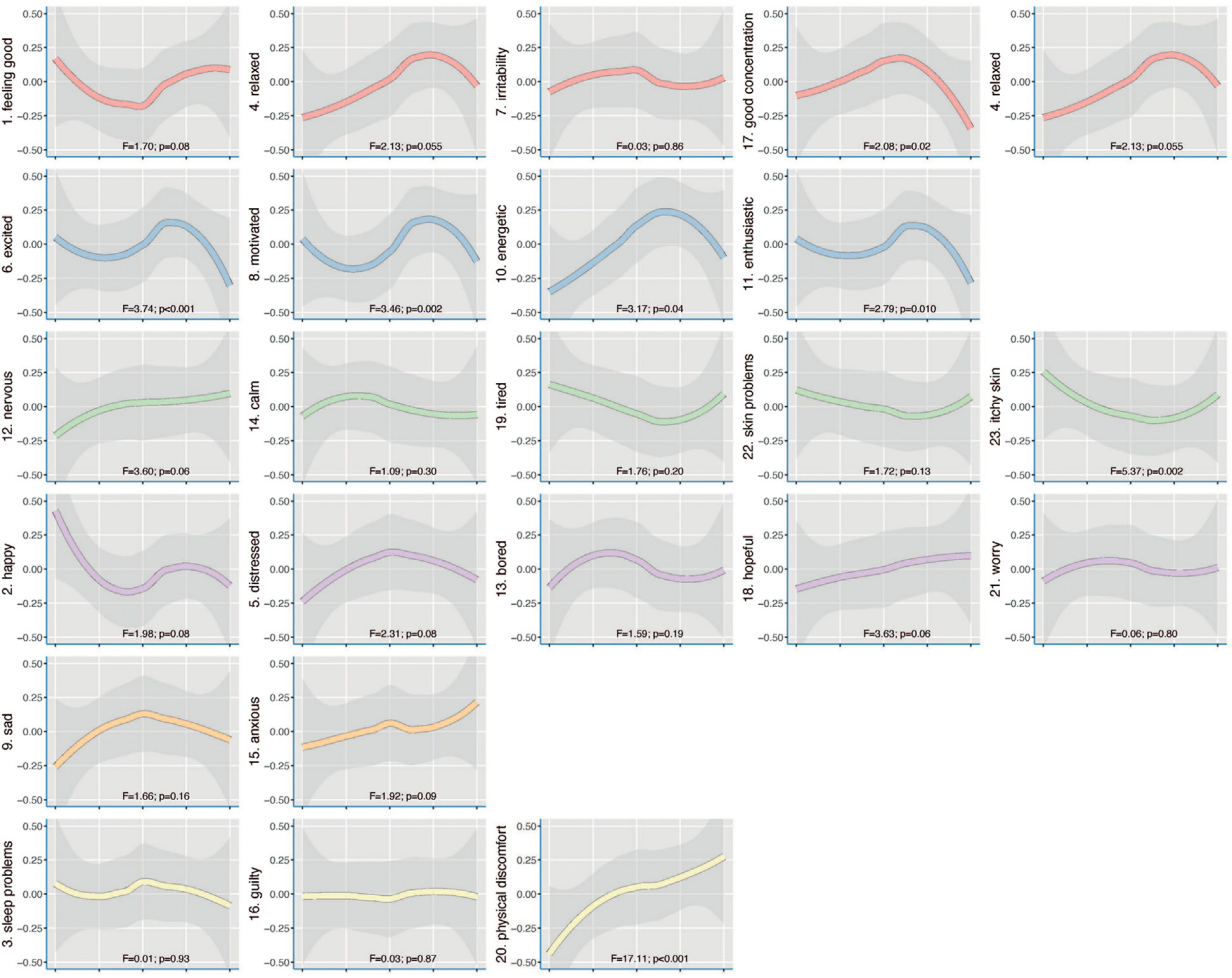
Average level of emotions and symptoms during glucocorticoid treatment (with the emotions colored according to the nomothetic symptom dimensions shown in Fig 3). The average level is shown with *thick lines*, with its 95% CI in *gray* surround it.

The generalized additive models modeled levels of the emotions and symptoms over time are shown in Figure 1. During glucocorticoid treatment positive psychological symptoms seemed to decline over time, eg, “excited,” “motivated,” “energetic,” “enthusiastic” (blue lines), and “good concentration” (red line). “Physical discomfort” (yellow line) seemed to worsen toward the end of treatment. Furthermore, the disease symptom “skin problems” appeared to remain quite stable during the treatment, and “itchy skin” (green lines) seemed to improve at the start of treatment, but worsened toward the end.

### Individual patient analyses (idiographic approach)

**Fig 2:**
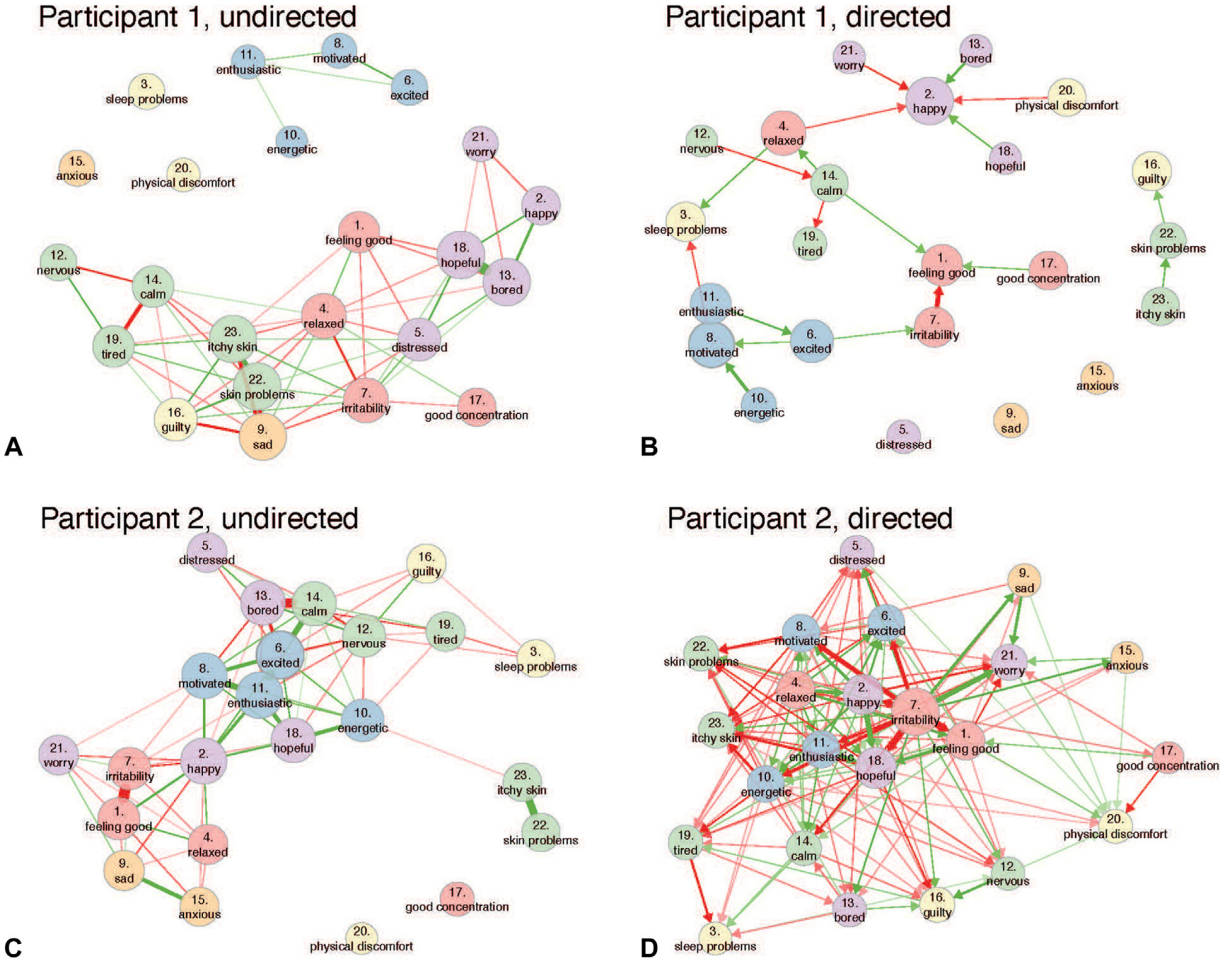
Idiographic dynamic time warp analysis. The directed and undirected networks of 2 participants are shown. A high degree of interindividual variability is found.

In order to assess fluctuations in emotions and symptoms during glucocorticoid treatment, we performed undirected and directed DTW analyses. The individual networks varied strongly among the 5 participants, with some very dense networks and others with more loosely connected networks in both undirected and directed analyses (Supplementary Fig 1, available via Mendeley at https://data.mendeley.com/datasets/6s8b3k9brv/1). Figure 2 shows an example of 2 individuals’ undirected and directed networks. In the undirected networks, most emotions and skin issues were interconnected, suggesting shared temporal dynamics. Skin problems and itchy skin were consistently connected to each other, which also suggests similar temporal dynamics. In the directed symptom networks, emotions and skin problem items shared many connections, where emotions/skin issues precede or follow other emotions/skin issues the next day. For example, in participant 1 “itchy skin” precedes “skin problems” the next day, and “physical discomfort” decreased being “happy” the next day. As already mentioned above, the individual networks highly differ between the participants.

### Group-level analyses (nomothetic approach)

**Fig 3:**
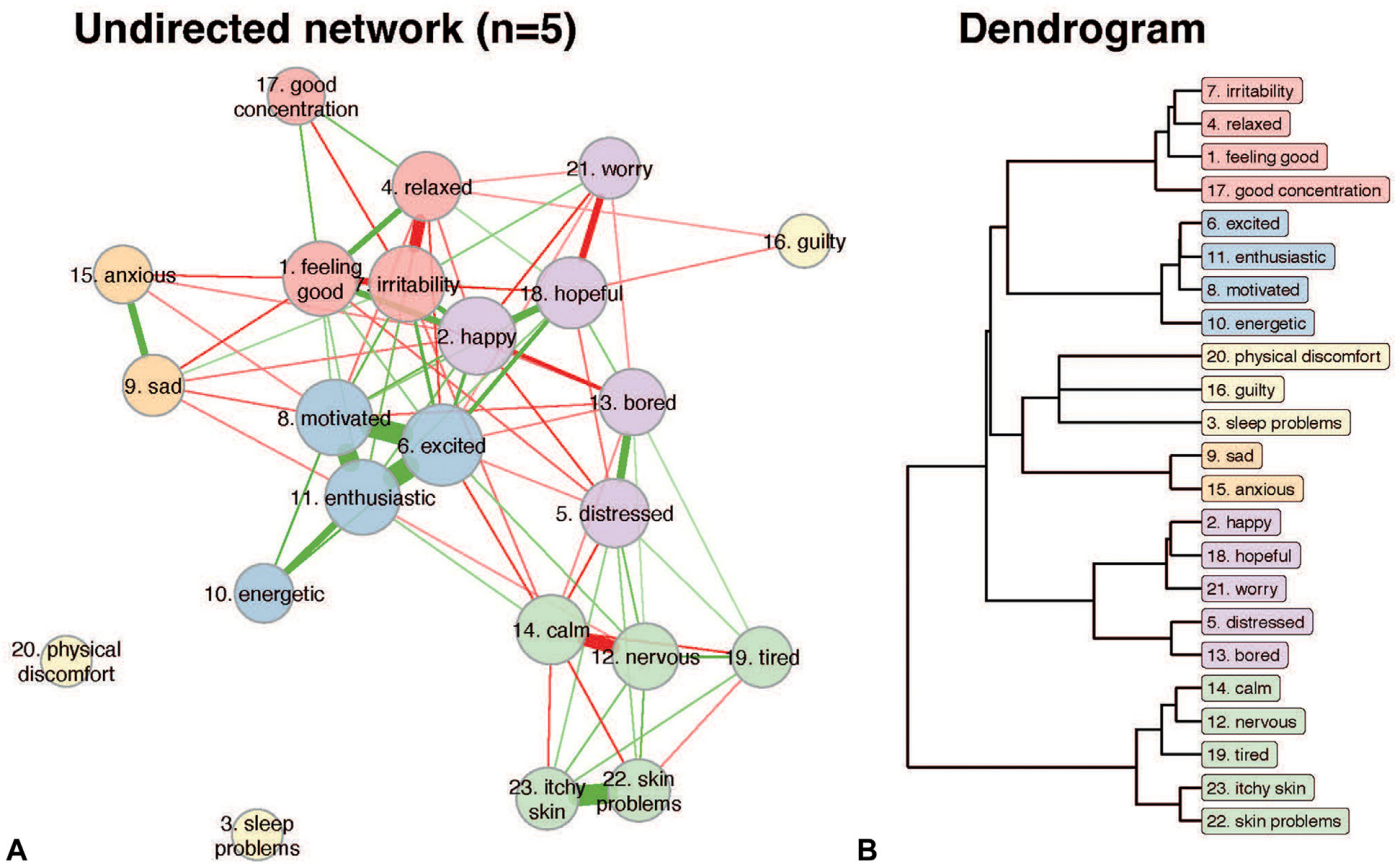
Undirected dynamic time warp analysis at the group-level. Panel **A** shows the undirected symptom network plot. The size of each node is proportional to the connectivity of that node: if an item shares many connections, this item node will be larger compared with an item with no or less connections. The nodes representing physical discomfort and sleep issues showed no significant connections to other nodes, suggesting their relatively independent dynamics over time. The edges are the lines connecting the nodes. The thickness of the edges is proportional to the strength of the undirected effect, with only edges that were significantly different from 0; *P* < .05 being shown. *Green edges* represent positive effects, *red edges* negative effects. Panel **B** shows the dendrogram of the clustering of emotions and symptoms with more similar trajectories over time. Each cluster is given a different color. So, all items from the same cluster share a similar color (also in panel **A**).

Figure 3 demonstrates the undirected DTW analyses of the 5 participants at the group-level. In the network, the skin problem items “itchy skin” and general “skin problems” were connected and exhibited the strongest connections to feelings of nervousness and a lack of calmness (Fig 3, *A*). Furthermore, the undirected network revealed 6 symptom clusters with similar trajectories (Fig 3, *B*). Cluster 1 included irritability, relaxed, feeling good, and good concentration. Cluster 2 included excited, enthusiastic, motivated, and energetic. Cluster 3 included physical discomfort, guilty, and sleep problems. Cluster 4 only consisted of sad and anxious, and cluster 5 included happy, hopeful, worry, distressed, and bored. Cluster 6 consisted of calm, nervous, tired, itch, and skin symptoms. The emotions within each cluster thus tended to behave similar over time.

**Fig 4:**
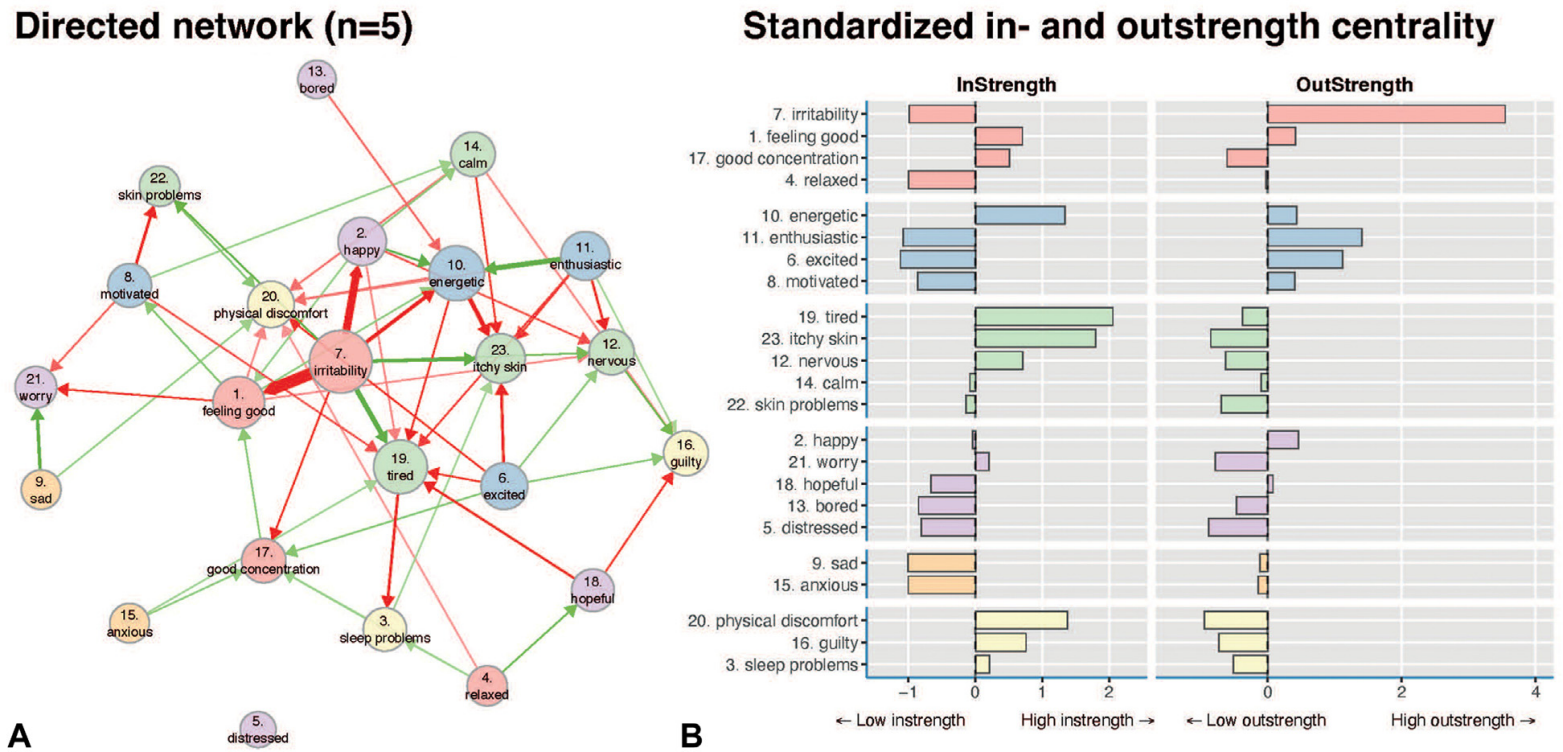
Directed dynamic time warp analysis at the group-level. Panel **A** shows the directed symptom network plot. In directed analysis, the flow is in one direction. The size of each node is proportional to the connectivity of that node: if an item shares many connections, this item node will be larger compared with an item with no or less connections. The edges are the lines/arrows connecting the nodes. The thickness of the edges is proportional to the strength of the directed effect, with only significant edges that were significantly different from 0; *P* < .05 being shown. *Green edges* represent positive effects, *red edges* negative effects. Panel **B** shows the in/outstrength values of the different emotions and symptoms. Items with many incoming edges will have high instrength, whereas items with many outgoing edges will have high outstrength. Irritability had the strongest outstrength level, ie, many outgoing edges. All items from the same cluster as shown in Figure 3, *B* share a similar color.

The directed DTW analyses at the group level revealed that irritability had the highest outstrength value (Fig 4). The high outstrength of irritability—despite its rather stable mean levels over time—implies that increasing or decreasing irritability tends to be followed by similar (negative) consequences the next day, such as increased itchiness, skin problems, and fatigue, and decreased concentration, well-being, energy, and happiness. Other emotions with high outstrength were enthusiastic and excited. There were also items with high instrength, eg, energetic, tired, physical discomfort, and itchy skin. This indicates that changes in these items tended to follow changes from other items from the previous day. The directed analyses also showed that irritability and experiencing low levels of positive emotions (such as feeling energetic, enthusiastic, and excited) were precursors of itchy skin. Similarly, irritability and low motivation were often followed by general skin problems.

## Discussion

In this proof-of-principle study, we demonstrated the potential of DTW as an analytical approach to investigate temporal relationships between emotions and disease-specific symptoms during glucocorticoid treatment in individual patients with CTCL. The average level of emotions, and disease-specific symptoms during glucocorticoid treatment, demonstrated that positive emotions tended to diminish over time. DTW analyses at the individual level, showed high variability in the symptom networks between participants. Despite the high variability at the individual level, undirected DTW group-level analysis yielded 6 symptom clusters with similar trajectories. Directed DTW group-level analysis showed high outstrength for irritability, enthusiasm, and feeling excited, suggesting that increased irritability, and decreased enthusiasm, and excitement tended to precede other emotions and skin problems the next day. The disease symptoms itchy skin and skin problems showed high instrength, indicating to follow upon low levels of positive emotions (such as feeling energetic, enthusiastic, and excited) the next day. Of course, the results of this proof-of-principle study need to be interpreted with care.

It is notable that despite the development of novel therapies, glucocorticoid therapy is still often prescribed for dermatologic, rheumatic, oncologic, and pulmonal diseases. Because of their frequent use, it is important to fully understand to potential negative effects on emotions as high dosage and long-term glucocorticoid treatment are associated with an increased risk of neuropsychiatric adverse effects.^1^^,^^2^ Although these more severe effects are recognized, the subtler implications of glucocorticoid therapy, which might be more common, have not been as extensively studied. Discontinuation of long-term glucocorticoid therapy is also associated with increased risk of depression and delirium/confusion,^22^ and glucocorticoid withdrawal symptoms can also occur after discontinuation of hormone therapy.^23^ Our observation that positive emotions diminish at the end of the glucocorticoid treatment, seems to complement those findings. Glucocorticoid use probably results in relative adrenal insufficiency, because the adrenal glands do not immediately resume normal cortisol production, not only inducing physical effects (eg, low blood pressure), but also diminishing positive emotions.

The high variability between the individuals was expected, and in line with findings of other studies using DTW in time-series data.^14^, ^15^, ^16^, ^17^, ^18^, ^19^ This variability is also consistent with the existing literature on glucocorticoid therapy, which is known to produce a wide range of side effects that vary greatly from person to person.^24^ Similar to these other studies, our data support the use of DTW in a personalized medicine setting. Patients differ in the emotions and symptoms they experience, and the reason why some patients do and others do not experience severe adverse effects is not understood yet. Network analyses have already shown that in dense networks, closely connected symptoms could cascade into activation of other symptoms, whereas in loosely connected networks this is less likely.^25^ Vulnerable networks with dense connections, could trigger the onset of severe adverse effects, whereas loose networks may make patients less vulnerable for developing these more severe adverse effects that affect whole clusters of symptoms.^26^ We have shown that with DTW, personalized symptom networks could be used to investigate the course of emotions and symptoms over time, and how they influence each other. Future studies with more participants, could focus on how glucocorticoids affect (individual) networks, and whether—or when—neuropsychiatric adverse effects are developed.

Irritability, enthusiastic, and excited were key emotions with the highest outstrength at the group level, implying that changes in these emotions tended to precede changes of other emotions the next day. Interestingly, irritability and mood swings are—among others—effects of glucocorticoids that matter to patients.^27^ Physicians could focus their intervention, treatment or counseling on key emotions and trying to prevent the triggering effects. Individual analyses in the current study revealed irritability as the key emotion in 2 participants. In these participant, mindfulness, relaxation, cognitive behavioral, and dialectic behavioral therapy may help to enhance skills to cope with feelings of irritability, anger and its autonomic dysregulations.^28^^,^^29^ Pharmacotherapy could also be considered, but only in case of severe and persisting symptoms. Antidepressants were found to be effective in the treatment of anger,^30^ whereas there is less evidence for effectiveness of sedatives.

We also found some interesting associations with skin problems and itchy skin. Feelings of nervousness and a lack of calmness covaried in time with these skin problem items. Despite the inclusion of only 5 participants, it is tempting to speculate that this may be a reflection of a bidirectional relationship: nervousness that may be triggered by glucocorticoids, potentially leading to scratching or picking at the skin,^31^ and vice versa nervousness and distress may activate the body’s stress response and worsens itch. Together with other stress responses, the glucocorticoids, although essential for primary cutaneous lymphoma treatment, may subsequently have influenced mood regulation, potentially impacting the manifestation of skin issues thereafter.

A limitation of our study is the inclusion of only 5 participants. Therefore, the group-level nomothetic analyses should be interpreted with care, and considered preliminary findings. One patient discontinued glucocorticoid treatment prematurely, and excluding this participant from group-level analyses affected group-level results. The latter demonstrates the vulnerability of group-level analysis, especially in diseases where symptoms very between individuals, such as mood or affective disorders. Not to mention the fact that emotions itself already vary among individuals. DTW analyses could help with more personalized treatment approaches, besides group-level results. Second, we did not include a control group. Future studies could investigate the temporal dynamics of emotions during glucocorticoid treatment by employing a larger sample size and using control groups to examine variations in emotional patterns with and without the use of glucocorticoids. In addition, maybe even a prediction could be made on which individuals are likely to develop symptoms during glucocorticoid usage based on their symptom network without glucocorticoid usage. Also, in the present study, participants did not report severe adverse effects on psychiatric symptoms, which likely is linked to the small number of subjects. Another limitation is our inability to disentangle the effect of the underlying disease from that of the glucocorticoid treatment on emotions. Also, the glucocorticoid treatment differed between the individuals, as the use of other therapeutics, which can influence the results.

A strength of the current proof-of-principle study is that it provides insights into temporal dynamics of emotions and disease-specific symptoms during a glucocorticoid treatment. We demonstrated the use of DTW as an analytical tool in capturing both individual, and group-level results. Another strength, is the frequent assessments per subject (on average 30 assessments); emotions and symptoms can change over the days, and with daily assessments these dynamics can be captured. It might also be interesting to extend the daily assessment to an even more frequent interval. However, too many assessments can increase the burden for the patients.^32^ Finally, both positive and negative emotions were included in the questionnaire.

To conclude, we demonstrated that glucocorticoid treatment seemed to coincide with decreased energy, motivation, excitement, and enthusiasm. Also, through directed group-level DTW analyses of ecological momentary assessment data, we found that irritability, and low levels of positive emotions were followed by skin problems and itchy skin the next day. All group-level findings are based on only 5 participants and are therefore preliminary and further research is needed to confirm and expand on these conclusions. DTW analysis could help with more personalized treatment approaches.

## Conflicts of interest

None disclosed.
